# Comparative outcomes of platelet concentrates and blood clot scaffolds for regenerative endodontic procedures: A systematic review of randomized controlled clinical trials

**DOI:** 10.4317/jced.60150

**Published:** 2023-03-01

**Authors:** Nestor Ríos-Osorio, Javier Caviedes-Bucheli, Oscar Jimenez-Peña, Mangriveth Orozco-Agudelo, Lorenzo Mosquera-Guevara, Fabio-Andrés Jiménez-Castellanos, Hernan-Darío Muñoz-Alvear

**Affiliations:** 1 DDS, MSc. Research Department COC- CICO, Institución Universitaria Colegios de Colombia UNICOC, Bogotá, Colombia; 2 DDS, MSc. Centro de Investigaciones Odontológicas Pontificia Universidad Javeriana Bogotá, Colombia; 3 DDS, MSc, PHD. Research Department COC- CICO, Institución Universitaria Colegios de Colombia UNICOC, Bogotá, Colombia; 4 DDS. Research Department COC- CICO, Institución Universitaria Colegios de Colombia UNICOC, Bogotá, Colombia; 5 DDS. Universidad Antonio Nariño UAN, Postgrado de periodoncia, Bogotá, Colombia; 6 DDS. Endodontics Department, Universidad Cooperativa de Colombia, Pasto- Colombia

## Abstract

**Background:**

The main objective of this systematic review is to evaluate the effectiveness of platelet concentrates -Platelet-rich plasma (PRP) or Fibrin-rich plasma (PRF)- compared with blood clot (BC) as scaffolds for maturogenesis, in patients with immature permanent teeth with or without AP, in terms of the criteria for pulp revascularization success.

**Material and Methods:**

We reviewed randomized controlled clinical trials comparing regenerative endodontic therapies (maturogenesis) based on PRP or PRF versus the conventional BC approach, in necrotic teeth with or without apical periodontitis (AP) under clinical and radiographic criteria. We performed a strategic search in MEDLINE (PUBMED), EMBASE, and ISI Web of Science from inception to October 2022. This systematic review of the literature was developed following the Cochrane Collaboration and PRISMA statement recommendations. We used the Cochrane risk of bias tool v2 to assess the included studies’ quality. We performed a qualitative synthesis of the evidence.

**Results:**

Ten randomized controlled clinical trials were included in this systematic review. Analyses of these studies suggest that maturogenesis is a successful therapy regardless of the method employed. However, further research should be conducted with more suitable research methodologies and more homogenous data for meta-analysis.

**Conclusions:**

Results from this systematic review suggest that BC maturogenesis approaches yield similar clinical and radiographic outcomes when compared to Platelet-concentrates based therapies (PRP and PRF).

** Key words:**Maturogenesis, Revascularization, Platelet-rich plasma, Fibrin-rich plasma, blood clot, systematic review.

## Introduction

Root formation proceeds under the control and influence of the Hertwig epithelial sheath and progenitor cells through epithelial-mesenchymal interactions (1). Root development interference can be related to several factors such as dentoalveolar injuries and infectious processes, which may lead to a loss of the neuro-vascular supply and subsequent development of periapical lesions, resulting in thin and fragile dentin root walls and absence of apical constriction (2). In such cases, the difficulty in generating an adequate apical seal put at risk the outcome of endodontic therapy (3).

Apexification has been traditionally the treatment of choice for necrotic immature permanent teeth with or without apical periodontitis (AP) (3). Apexification aims to establish an apical mechanical barrier of mineralized tissue in roots with immature apex (2,3). However, the result from this therapy is generally a biomechanically unsTable tooth, since canal walls remain thin and fragile, and root length short, as a consequence of the cessation of root formation (2,3).

A regenerative endodontic therapy (RET) commonly termed revascularization has also been recommended for treating such immature permanent teeth. RETs aim to restore the blood supply and enable continued root development, by replacing cells related to radicular morphogenesis and injured root tissues (4). Therefore, such therapies should be described as maturogenesis, since the term revascularization is imprecise as it only covers one aspect of RET (4,5).

Maturogenesis depends on three critical components: (i) Mesenchymal stem cells, which provide a source of primary odontoblasts-like cells (ii) Signaling molecules for mesenchymal stem cells stimulation, differentiation and proliferation, and (iii) a Physical scaffold for providing a suitable environment for cells responsible for continuing root development (4-7).

The conventional bleeding technique of maturogenesis involves minimal mechanical instrumentation, root canal irrigation with sodium hypochlorite (NaOCl), and the use of an intracanal tri-antibiotic paste ( Metronidazole, Ciprofloxacin, and Minocycline), followed by laceration of the periapical tissues aimed at stabilizing a blood clot (BC) scaffold within the immature root canal (4,5). The BC creates a three-dimensional scaffold that entraps undifferentiated mesenchymal stem cells. The platelets and fibrin-rich plasma within the BC contain bioactive signalling molecules which can interact with the mesenchymal stem cells, thus inducing tissue regeneration (4,5).

Currently, some bioactive autologous derivatives (platelet concentrates) such as platelet-rich plasma (PRP), and fibrin-rich plasma (PRF) have been proposed as a new alternative to replace BC scaffolds for maturogenesis. (6).

PRP is an autologous scaffold rich in growth factors, obtained by platelet activation and fibrinogen polymerization. PRP promotes cell differentiation, collagen production and angiogenesis, in addition to possessing anti-inflammatory and anti-bacterial properties (8). PRF, the second generation of these platelet concentrates may boost migration and cellular activity, as, during any hemostatic and healing phenomenon, the fibrin clot traps the stem cells which are directed towards the wound site (8). Therefore, it is conceivable that the usage of platelet concentrates could improve and speed up tissue regeneration processes.

Platelet concentrates represent a new option in the field of RETs, however, comparative outcomes Vs. Conventional BC maturogenesis approaches remain poorly studied. In addition, previous studies addressing this topic have shown inconsistent results and methodological flaws (9). Consequently, this study aims to perform a systematic literature review to evaluate the effectiveness of platelet concentrates (PRP and PRF) compared with BC as scaffolds for maturogenesis in patients with immature permanent teeth with or without AP.

## Material and Methods

We strictly followed the Cochrane Collaboration and PRISMA statement recommendations (10). A detailed protocol was developed and registered in PROSPERO under the ID: CRD42022363810.

Criteria for regenerative endodontic therapy success.

The American Association of Endodontics AAE defined three main goals that should be archived to consider a regenerative endodontic procedure as successful: (i) Resolution of clinical signs and symptoms, and evidence of bone healing, (ii) continued root development in terms of increased root wall thickness and/or increased root length, and (iii) Positive response to vitality tests (11).

PICO question: Using the PICO strategy, the focused question and the inclusion criteria were framed.

Population: Permanent immature necrotic teeth with or without AP. Intervention: PRF or PRP as scaffolds for maturogenesis. Comparison: Blood clot scaffolds. Outcome: Increase in root length, thickening of dentinal walls, evidence of apical bone healing and positive response to vitality tests.

Focused question: Do platelet concentrates (PRP and PRF) improve the outcomes of maturogenesis therapies when compared with BC scaffolds?

Eligibility

Inclusion criteria: We included randomized controlled clinical trials comparing directly (PRP or PRF) Vs. BC as scaffolds in regenerative endodontic treatments of immature permanent teeth with necrotic pulp with or without AP under clinical and radiographic criteria with at least a one-year follow-up period (12 months).

Exclusion criteria: We excluded studies that did not define the evaluation method, In vitro or animal studies, studies that did not compare PRP or PRF directly with BC maturogenesis techniques, and studies that did not clearly define the maturogenesis protocol.

Information sources

We searched MEDLINE (PUBMED), EMBASE, and ISI Web of Science from inception to October 2022. References from relevant articles identified through the search, open grey, thesis databases, clinicaltrials.gov and Google scholar among others were also scanned. The next search strategy translated for each database:

“(endodontics AND revascularization OR regenerative endodontic procedure OR regenerative endodontic treatment OR pulp revitalization AND necrotic dental pulp AND necrotic permanent teeth AND necrotic teeth AND immature permanent teeth) AND (platelet rich plasma OR platelet rich fibrin OR blood clot)”.

Data collection

Two researchers reviewed independently each reference by title and abstract. Full texts of relevant studies were scanned to apply specific inclusion and exclusion criteria, and finally, the data was extracted. Disagreements were resolved by consensus, otherwise, a third reviewer solved the disagreements. Two calibrated reviewers working independently, extracted the following information from each reference: title, year of the publication, author’s names, study design, objectives, inclusion and exclusion criteria, number of patients included, sample characteristics losses to follow up, outcomes and association measures and geographic location.

Risk of bias

Risk of bias was assessed for the studies Included in qualitative synthesis with the Cochrane Risk of Bias tool (version 2) for clinical trials.

Data analysis

We could not conduct a meta-analysis since the included articles in this systematic review were methodologically heterogeneous. Therefore, comparisons between interventions could not be performed .

## Results

-Study selection 

**Figure 1:**
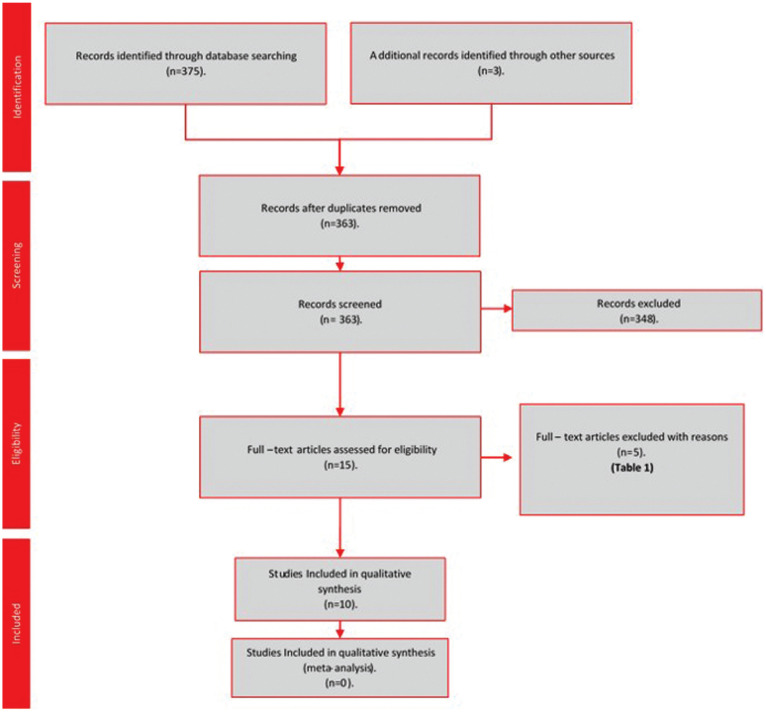
Flowchart of included studies.

378 references were initially identified through the search strategy. After the removal of 15 duplicates, we screened 363 titles/abstracts. 15 Full – text articles were assessed for eligibility. After full article screening, 5 references were excluded with reasons (12-16) (Table 1). Finally, ten randomized controlled clinical trials met the inclusion criteria for qualitative synthesis (6,17-25) (Fig. 1).

**Table 1:**
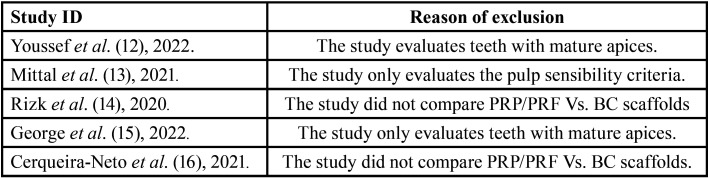
Full–text articles excluded with reasons.

-Characteristics of included studies

Ten studies were included in this systematic review (6,17-25), published between 2012 and 2021 (eight studies (6,17,19-24), compared BC Vs. PRP and five studies (17,18,21,22,25), compared BC Vs. PRF). The included studies were conducted in India (6,21,22,24), Egypt (18,23,25) Saudi Arabia (20) and Turkey (17,19). All the studies were randomized controlled clinical trials, with at least a 12-month (one-year) follow-up period. Two studies with an 18-month follow-up period (19,21), and one study with a follow-up for a period ranging from 10-49 months (17). The age of the participants in these studies ranged from 7 to 54 years. When treated, all teeth were immature and diagnosed with pulp necrosis with or without AP. Analysis of the ten randomized clinical trials included in this systematic review suggests that maturogenesis is a highly predictable practice, regardless of the scaffold used (BC/PRP/PRF). None of the scaffolds analyzed in this review substantially influenced the outcomes of maturogenesis. Treatment outcomes did not differ significantly between scaffolds. Notably, although the European society of endodontics and the American Association of Endodontics have recommended standardized protocols (26,27), most of the included studies applied different protocols regarding intracanal irrigation and the composition of the tri-antibiotic paste. The language of the publication of all studies was English (Table 2).

**Table 2:**
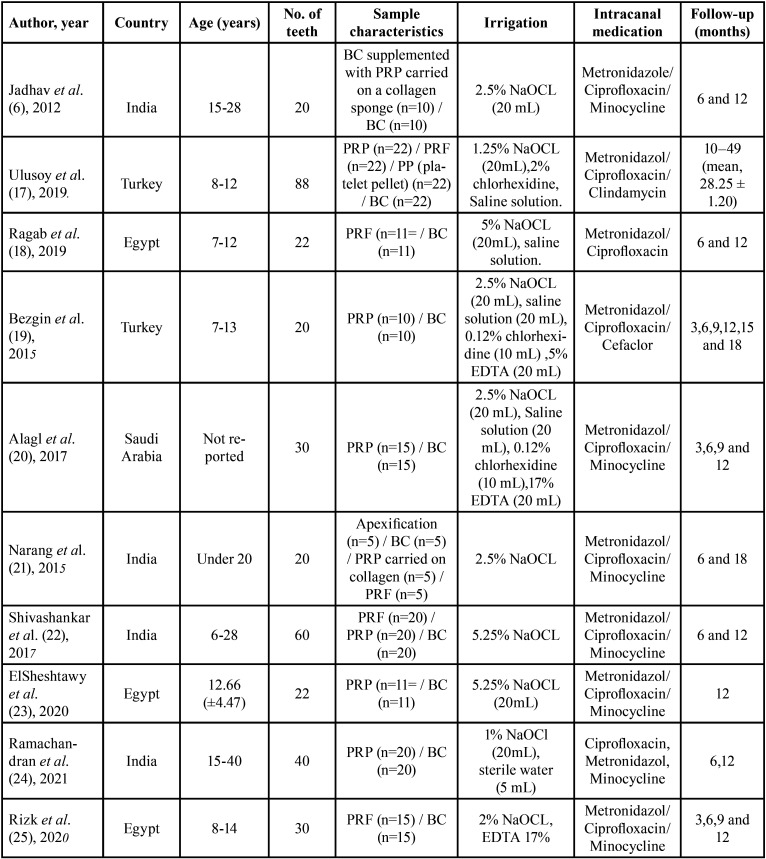
Characteristics of the included studies.

-Risk of bias assessment 

**Figure 2:**
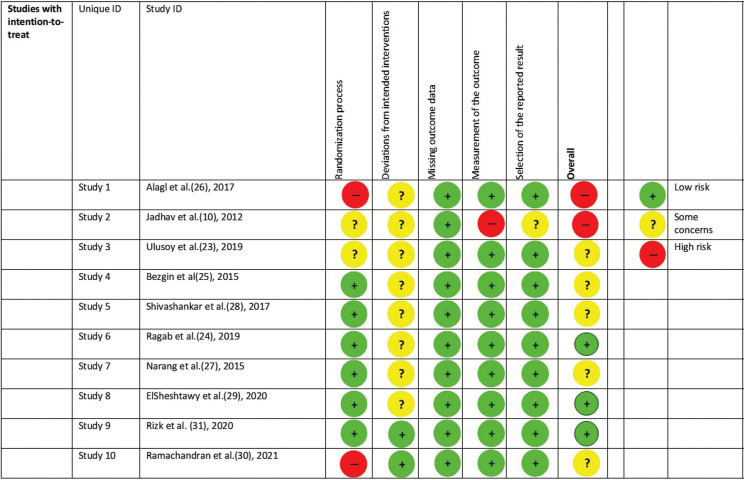
Risk of bias assessment.

Regarding the randomization process, two studies showed some concerns (Jadhav *et al*. 2012 and Ulusoy *et al*. 2019) and other two (Ramachandran *et al*. 2021 and Alagl *et al*. 2017) presented high risk (6,8,20,24). Jadhav *et al*. 2012, also showed some concerns regarding deviation from standard interventions and the selection of the reported results and high risk regarding the measurement of the outcome (6). Alagl *et al*. 2017, also showed some concerns regarding deviation from standard interventions (20). Jadhav *et al*. 2012 and Alagl *et al*. 2017, were rated as at high risk in the overall result (6,20). Eight studies showed a deviation from standard interventions (6,17-23). Finally, three out of the ten selected studies (Ragab *et al*. 2019, Elsheshtawy *et al*. 2020 and Risk *et al*. 2020), had an overall low risk of bias (18,23,25) (Fig. 2).

-Synthesis of the evidence 

The ten randomized controlled clinical trials included in this systematic review demonstrated unanimity in terms of satisfactory clinical outcomes (eliminations of clinical signs and symptoms related to pulp necrosis and AP, such as pain, abscess and/or fistula, and sensitivity to percussion and palpation) after carrying out any of the three evaluated maturogenesis protocols (BC, PRP or PRF). During the entire course of the follow-up periods of the evaluated studies (6,17-25), most of the patients were asymptomatic. Two patients (one treated with the PRF approach and one treated with the BC approach) from one study showed clinical signs and symptoms of endodontic failure (17). Another study reported that two participants in the BC group and one participant in the PRP group had signs of re-infection (23). Finally, four patients were considered as failed because of the presence of pain (24). Likewise, the radiographic analysis revealed that most of the patients included in the studies (6,17-25) showed some degree of radiographic root development and periapical healing in cases of AP, regardless of the employed maturogenesis technique.

-BC Vs. PRP scaffolds

Bezgin *et al*. (19) reported that teeth treated with the BC approach exhibited a mean increase of 12.6% in the root area, compared with 9.86% in the PRP approach (*P* > 0 .05). There were also no statistically significant differences (*P* >0 .05) in terms of healing time according to lesion size and positive response to vitality tests. Likewise, the time required to obtain a complete apical closure was also similar between groups (a mean of 8.1 months in the PRP group / nine months in the BC group) (19). Alagl *et al*. (20), reported similar results in terms of periapical healing, apical closure, and positive response to pulpal sensitivity testing. only the mean difference in the root length was found to be statistically significant in the PRP group when compared with the BC group (*P* < .004). The authors suggest that PRP alone cannot significantly affect maturogenesis outcomes (20). Likewise, Elsheshtawy *et al*. (23) reported that changes in root wall thickness, root length, apical closure and radiographic root area were found to be significant for both groups (PRP and BC), but, without differences between both groups (*P* >0 .05) (23). Recently, Ramachandran *et al*. (24) compare PRP Vs. BC scaffolds in terms of change in the radiographic root area. This study also concluded that there was no difference (*P* >0 .05), in the percentage change in the root area between both groups after a 1-year follow-up period (24). Finally, one study by Jadhav *et al*. (6) found statistically significant differences (*P* <0.05) between the PRP group and the BC group in terms of periapical healing, apical closure, and dentinal wall thickening, suggesting that maturogenesis using the PRP approach potentially improves the desired biological outcomes of maturogenesis (6).

BC Vs. PRF scaffolds

Narang *et al*. (21) compared the regenerative potential of the three methods (PRF, PRP, and BC). The authors reported that the PRF group showed a statistically significant difference (*p* ˂0.05), regarding periapical healing (*P* = 0.003), root lengthening (*P* = 0.002) and dentinal wall thickening (*P* = 0.047). These results suggest that PRF favours most of the desired biological outcomes of maturogenesis when compared to PRP and BC (21). comparable findings were published by Rizk *et al*. (25) in a split-mouth double-blind parallel arm trial, who reported that PRF showed statistical significance (*p* ˂0.05) in terms of increase in root length, wall thickening, periapical healing, and reduction in apical diameter when compared with the BC group at all follow-up periods (25).

Contradictory results were reported by Ragab *et al*. (18) who evaluated the effect of BC and PRF in terms of root lengthening and periapical healing after a one-year follow-up period. There were no statistically significant differences (*p*>0.05) between the two groups (18). Likewise, Shivashankar *et al*. (22) who compared PRF, BC and PRP, reported that there was no significant difference (*p*>0.05) among the three groups concerning dentinal wall thickening and response to vitality testing (22). Similar results were reported by Ulusoy *et al*. (17) who evaluated 77 patients with 88 immature necrotic incisors (randomly assigned into four different groups: PRP, PRF, platelet pellet (PP), and BC). 73.9% of all the evaluated teeth showed complete apical closure after the follow-up period (28.25 ±1.20 months) with no statistically significant differences among groups (*p*> 0.05). Likewise, linear measurements indicated a similar increase in terms of periapical healing, root length and width among all groups (*P* > 0.05). The study also showed similar results in terms of response to pulp testing among the treatment groups (*p*>0.05). The authors concluded that the “BC scaffolds may yield similar clinical and radiographic outcomes to PRP and PRF” (17) (Table 3,  Table 3 cont.).

**Table 3:**
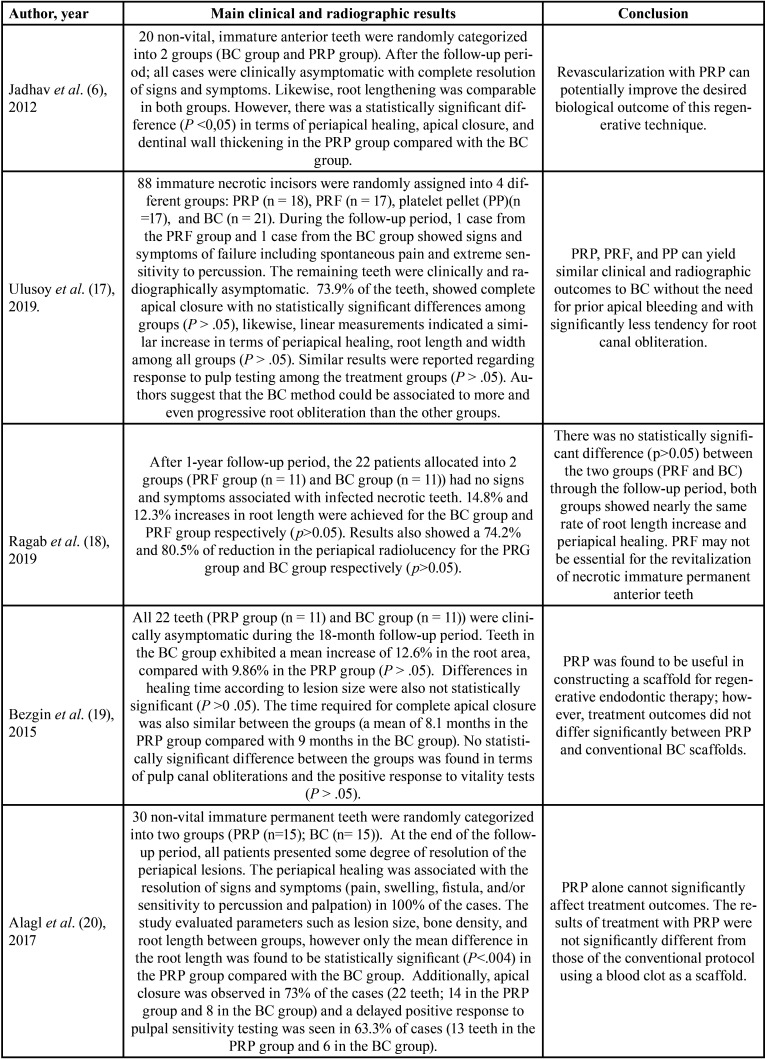
Description of the main outcomes.

**Table 3 cont.**
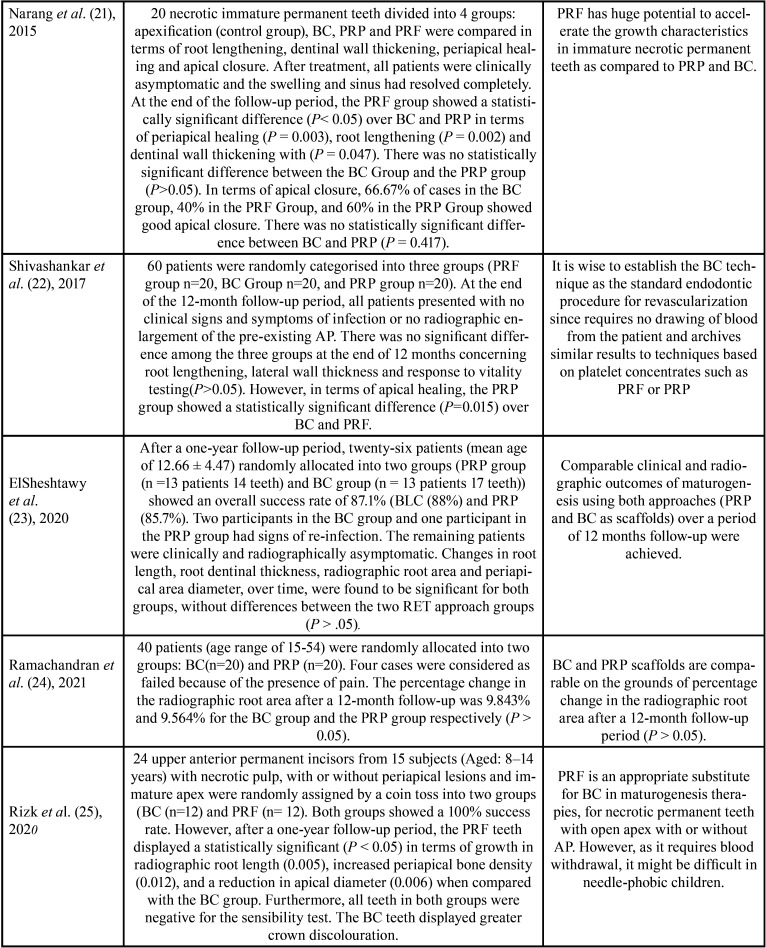
Description of the main outcomes.

## Discussion

RETs were successful clinically and radiographically in all the randomized controlled clinical trials included in this systematic review (6,17-27), regardless of the employed technique (BC, PRP, PRF), since most of the patients showed resolution of clinical signs and symptoms, presence of some degree of periapical healing, increase in root length and root thickness and apical closure. As a result of the risk bias evaluation, two studies (6,20) were classified as high risk of bias. Five studies (17,19,21,22,24) were rated as at some concerns, and three studies were classified as low risk of bias (18,23,25). In general terms, this systematic review presents a moderate risk of bias.

Nygaard-Ostby (1961), reported that a BC inside a root canal, created by intentionally lacerating the periapical tissues is gradually replaced by the ingrowth of granulation tissue, which in turn gives rise to fibrous connective tissue (28). In a later study, Nygaard-Ostby and Hjortdal (1971), demonstrated the deposition of cellular cementum within root canals partly filled with a BC (29). More recently, it has been reported that the tissue forming inside the root canal system after maturogenesis combines a fibrous connective tissue and bone-like substance with vascular-like structures (30). Studies have also indicated that this tissue may help the innate immune system reappear, which could prevent root canal system reinfection (31).

Maturogenesis studies utilizing BC as a scaffold have reported high success rates ranging from 90% to 94% (21,32,33). However, the use of BC scaffold for maturogenesis is still a concern, as it is likely to evoke tissue healing rather than pulpal regeneration (23). On the other hand, failure to provoke apical bleeding or to achieve adequate blood volume within the necrotic root canal and the discomfort caused by the mechanical irritation of periapical tissues also remain concerns (17).

PRP and PRF have been proposed as ideal scaffolds for RET (6,21,25). A human blood clot contains only 5% platelets, whereas, a PRP clot contains about 95%; therefore, platelet concentration may increase up to 740% within a PRP clot (21). Likewise, PRF contains a 210-fold higher concentration of platelets when compared to a human BC (21). Accordingly, it could be thought that tissue regeneration processes could be accelerated when platelet concentrates are used. However, results from 7 out of 10 studies included in this systematic review failed to show superiority of the platelet concentrates (PRP and PRF) over the BC approach in terms of clinical and radiographic outcomes (17-20,22-24). Although, such results could be associated with the relatively short follow-up periods, lack of radiograph and image standardization and calibration across the trials and the shortcomings regarding the risk of bias of the included studies.

PRP elicit a sustained release of growth factors that boost undifferentiated mesenchymal stem cells, which in turn stimulates the production of collagen and local anti-inflammatory agents such as RANTES/CCL5, thus improving soft- and hard-tissue regeneration (6). However, only one study (6) out of the eight (6,17,19-24), included in this systematic review that directly compared PRP vs. BC, concluded that maturogenesis with PRP could improve the desired biological outcomes (6). Notably, it should be noted that in that study, the PRP was used as a supplementation to the BC approach and never applied alone (6). The remaining seven studies (17,19-24), concluded that both clinical and radiographic outcomes did not differ significantly. Histologic studies have suggested that PRP alone does not significantly affect RET outcomes (34,35). Furthermore, Martin *et al*. (2013), observed that the tissues formed inside the root canals after carrying out a maturogenesis protocol either with BC or PRP are similar histologically (34).

PRF has several advantages over PRP. PRF comprises only autologous components, thus being more suiTable for growth factors storage and cell migration (17). Moreover, PRF performs a slower sustained release of the stored growth factors such as the Platelet-derived growth factor (PDGF) and the Transforming growth factor-beta (TGF-β) and its dissolution is longer after application, as it remodels similarly to a natural blood clot (21,36). Unlike PRP, PRF boosts bone undifferentiated mesenchymal cells, enhancing their proliferation and differentiation (36). However, only two studies (21,25), out of five (17,18,21,22,25), included in this systematic review that directly compared PRF vs. BC, reported that PRF showed a statistically significant difference (*p*<0.05) over BC in terms of periapical healing, root lengthening, dentinal wall thickening and decrease in apical diameter. Results from the remaining 3 studies (17,18,22), showed that both approaches could yield similar clinical and radiographic outcomes. This may suggest that a BC formation and stabilization inside an empty root canal may be an ideal scaffold for grown factors and stem cells deriving from the apical papilla. Stem cells deriving from the apical papilla survive pulp necrosis even in the presence of periapical infection and provide a source of odontoblasts like-cells (4).

On the other hand, Ulusoy *et al*. (17) reported that, although there were no significant differences (*p*>0.05) among the different maturogenesis approaches (BC, PRP and PRF) regarding most of the evaluated parameters, pulp maturogenesis with PRP and PRF showed significantly faster initial response to vitality test than the BC group, which may indicate a higher degree of organization of the vital pulp tissue (17). These findings could be associated with a higher platelet concentration in PRP and PRF compared to BC, which in turn can be related to a higher capacity for sensory fibre regeneration (17). Notably, “the lack of a pulp response does not necessarily indicate a lack of vitality” (37).

Another possible disadvantage of BC scaffold is its higher propensity for canal obliteration than PRP and PRF, which can become a complication in the case of requiring endodontic therapy in the future (17). Such obliterations may be related to the apical bleeding induced in the BC method, which may carry non–stem cells from the apical papilla that could elicit an ectopic apposition of mineralized tissues on the root canal walls (17). However, there is not enough evidence to support endodontic therapy in the case of mineralized obliteration unless AP is observed or the obliterated tooth becomes symptomatic (19).

Finally, the root canal microbiome must be efficiently controlled to allow the regeneration of periapical tissues. This process is the key to achieving long-term success in REP. Therefore, in observing the successful clinical and radiographic results, it must be considered; besides the maturogenesis approach used, the previous intra-canal antibiotic therapy employed. The included studies in this systematic review described that the composition of the intra-canal antibiotic ranged among the following combinations: Metronidazole and ciprofloxacin (18), metronidazole, ciprofloxacin, and cefaclor (19), metronidazole, ciprofloxacin, and minocycline (6,20,22), and clindamycin, ciprofloxacin and Metronidazole (17).

Hoshino *et al*. (38) demonstrated the antimicrobial efficiency of a triple-antibiotic paste in the composition of metronidazole, ciprofloxacin, and minocycline (38). A combination of antibiotics should be used to address the polymicrobial nature of endodontic infections and reduce the likelihood of developing resistant bacterial strains (38). Recent studies have reported success in maturogenesis with cefaclor and clindamycin used in place of minocycline in the triple antibiotic paste, or just by omitting the use of minocycline to avoid tooth discolouration (17-19). Another possible drawback of intracanal antibiotics besides tooth discolouration is their detrimental effect on stem cell survival. It has been reported that different combinations of intra- canal antibiotics risk the survival of human apical papilla stem cells (39). However, the toxicity to stem cells can be avoided by using concentrations below 1 mg / mL (39).

In view of the importance of maintaining the integrity of periodontal ligament cells for revascularization repair, recent studies have suggested the use of calcium hydroxide, alone or in combination with chlorhexidine, as intracanal medication in place of tri-antibiotic paste and have reported successful results in terms of clinical and radiographic outcomes, comparable with results obtained with approaches using tri-antibiotic paste (40). However, no study using calcium hydroxide as intracanal medication was included in this systematic review, since none met the inclusion criteria during the screening.

Knowledge regarding the nature of the resulting tissues following a RET is fundamental in estimating tooth survival and treatment prognosis (37). However, up to date, “there is a lack of histological and biomolecular data on the tissues responsible for apical closure, canal narrowing and even the recovery of pulp sensibility” following a RET (37). Therefore, the results of this systematic review should be analyzed with caution since the studies included in this study did not provide direct evidence for repair or regeneration at the histologic level. Furthermore, the findings of this systematic review demonstrated a scarcity of randomized controlled clinical trials comparing regenerative endodontic therapies based on platelet concentrates vs. BC approaches. Moreover, most of the included articles in this systematic review were methodologically heterogeneous and had shortcomings regarding the risk of bias. Therefore, more clinical investigations evaluating the effectiveness of platelet concentrates Vs. the conventional BC maturogenesis technique in patients with immature permanent teeth with necrotic pulp with or without AP should be conducted with more suiTable research methodologies and more homogenous data for meta-analysis.

## Conclusions

Maturogenesis can be considered a successful therapy regardless of the method used (BC, PRP, and PRF). However, there is a need for better-designed studies describing long-term outcomes. Platelet concentrates (PRP and PRF) cannot be suggested to be superior to BC methods in terms of the criteria for pulp maturogenesis success (clinical and radiographic outcomes). Results from this systematic review suggest that the BC maturogenesis approach yield similar clinical and radiographic outcomes when compared to Platelet-concentrates based therapies.
